# PPARs and Bone Metabolism

**DOI:** 10.1155/PPAR/2006/18089

**Published:** 2006-09-06

**Authors:** Beata Lecka-Czernik

**Affiliations:** Department of Geriatrics, Reynolds Institute on Aging, University of Arkansas for Medical Sciences, Little Rock, AR 72205, USA

Welcome to the inaugural *special* issue of *PPAR Research*: *PPARs and Bone Metabolism*. In addition to the key roles PPARs play in numerous processes including glucose and fat metabolism, inflammation, cancer, and central nervous system maintenance, a new role for PPAR-γ has recently emerged: the maintenance of bone homeostasis during aging and disease. In this premier issue we have assembled what is close to a comprehensive overview of the role of PPAR-γ in the control of bone maintenance. This takes into account PPAR-γ's role in mesenchymal stem cell lineage allocation, possible cross-talk with relevant nuclear receptors, examination of PPAR-γ gene polymorphisms and bone mineral density in humans, a role of PPAR-γ in bone loss due to skeletal disuse, evidence that human bone is vulnerable to antidiabetic therapies with PPAR-γ agonists, the thiazolidinediones, and evidence that the antiosteoblastic activity of PPAR-γ can be separated from its proadipocytic and antidiabetic activities by using selective modulators. We also present a novel hypothesis that PPAR-γ acts as a regulator of chondrocyte development and cartilage homeostasis. We realize that we have not covered all aspects of PPARs involvement in the control of bone maintenance; however, this introduction should serve as a competent first attempt to present these new aspects of bone biology to the broader audience of our readers.

*Beata Lecka-Czernik* *Beata Lecka-Czernik*

